# Unavailability of liver triacylglycerol increases serum cholesterol concentration induced by dietary cholesterol in exogenously hypercholesterolemic (ExHC) rats

**DOI:** 10.1186/1476-511X-13-19

**Published:** 2014-01-22

**Authors:** Yasutake Tanaka, Koji Nagao, Hideaki Nakagiri, Toshirou Nagaso, Yasue Iwasa, Haruhiko Mori, Makoto Asahina, Katsumi Imaizumi, Masao Sato

**Affiliations:** 1 Laboratory of Nutrition Chemistry, Division of Bioresource and Bioenvironmental Sciences, Graduate School, Kyushu University, 6-10-1 hakozaki, higashi-ku, Fukuoka 812-8581, Japan

**Keywords:** Hypercholesterolemia, ExHC rats, Triacylglycerol, Fatty acid synthase, Golgi apparatus, β-VLDL

## Abstract

**Background:**

Exogenously hypercholesterolemic (ExHC) rats develop hypercholesterolemia and low hepatic triacylglycerol (TAG) levels when dietary cholesterol is loaded. The responsible gene *Smek2* was identified via linkage analysis using the original strain Sprague–Dawley (SD) rats. In this study, we compared SD and ExHC rats to investigate a relationship between hypercholesterolemia and the low hepatic TAG levels observed in ExHC rats.

**Methods:**

Male 4-weeks-old ExHC and SD rats were fed a 1% cholesterol diet for 1 week. Serum and liver parameters were analyzed. Gene expression and enzyme activities related to TAG metabolism were also assessed.

**Results:**

We reproducibly observed higher serum cholesterol and lower hepatic TAG levels in ExHC rats than in SD rats. Golgi apparatus in the livers of ExHC rats secreted β-very-low-density lipoprotein (β-VLDL) that had higher cholesterol ester (CE) and lower TAG content than those in the β-VLDL secreted by SD rats. Gene expression related to fatty acid and TAG synthesis in ExHC rats was lower than that in SD rats. Enzymatic activities for fatty acid synthesis were also relatively lower in ExHC rats. Moreover, the fatty acid composition of hepatic and serum CE in ExHC rats showed that these CEs were not modified after secretion from the liver despite the similar activities of serum lecithin-cholesterol acyltransferase (LCAT) in ExHC rats to those in SD rats.

**Conclusions:**

Low production of liver TAG and secretion of CE-rich, TAG-poor β-VLDL without modification by LCAT in the circulation contributed to hypercholesterolemia induced by dietary cholesterol in ExHC rats.

## Introduction

For decades, high serum total cholesterol has been identified as a clinical risk factor for atherosclerosis and cardiovascular disease [1,2]. The factors that determine serum total cholesterol levels are cholesterol synthesis in the body, which is controlled by genetic expression [3,4], and daily dietary intake [5,6]. Gene mutations related to cholesterol metabolism have been detected mainly in the region involved in serum lipoprotein metabolism [7]. As compositive matters of these factors, hyperresponders to dietary cholesterol, in whom serum cholesterol levels increase more than those of normal subjects after a high-cholesterol-diet period, have been reported by Katan et al. [8]. However, the causes and mechanisms for regulating sensitivity to dietary cholesterol remain unclear. Exogenously hypercholesterolemic (ExHC) rats were established from Sprague–Dawley (SD) rats as models for diet-induced hypercholesterolemia [9]. Their serum total cholesterol levels are increased only when fed a cholesterol diet. ExHC rats do not develop aortic atherosclerosis despite attempts to establish a model rat for atherosclerosis [9]. Clarification of responsible genes and pathogenic mechanisms in ExHC rats will contribute to understanding the relationship between sensitivity to dietary cholesterol and hypercholesterolemia.

Several methods, including sib-pair analysis in human populations [10], linkage mapping analysis in animal models, and cholesterol-metabolic speculations [11], have been used to scan responsible genes for determinants of serum cholesterol concentration. We previously performed linkage analysis adapted to ExHC rats and found *Smek2* as a gene responsible for dietary cholesterol-induced hypercholesterolemia [12]. With this analysis, we established homocongenic Ex.BN-Dihc2 rats (congenic rats), in which the responsible region for diet-induced hypercholesterolemia on chromosome 14 was recombined to a genome derived from brown Norway (BN) rats. Compared with ExHC rats, congenic rats showed significantly higher hepatic triacylglycerol (TAG) content as well as significantly lower serum cholesterol levels [12]. However, the roles of *Smek2* in lipid metabolism have not been defined. One report has implicated *Smek2* as well as *Smek1* in the regulation of gluconeogenesis [13]. Therefore, we investigated whether ExHC rats had lipid metabolism phenotypes that displayed known roles of *Smek2*. ExHC rats, as phenotypes, display dietary cholesterol absorption and transport, hepatic cholesterol synthesis, and secretion similar to those of SD rats, which were fed a cholesterol-containing diet [14]. Liver cholesterol content in ExHC rats is identical to that in SD rats, but TAG content in the liver is significantly lower in ExHC rats than in SD rats [15]. Moreover, cholesterol secretion in the range of d < 1.006 g/mL lipoproteins did not differ between the two strains, whereas TAG secretion levels in the range of d < 1.006 g/mL lipoproteins were significantly lower in ExHC rats [14]. Compared with SD rats, ExHC rats show decreased hepatic uptake of serum cholesterol [14]; the rate of bile acid secretion into feces is similar between the strains [14]. In general, cholesterol intestinal absorption [14], hepatic synthesis [14], and excretion in feces [15] in ExHC rats are similar to those in SD rats.

As described above, ExHC rats do not display typical abnormalities in cholesterol metabolism. To elucidate the mechanism underlying the increase in serum cholesterol induced by dietary cholesterol in ExHC rats, we first investigated lipid metabolism in the liver, especially TAG metabolism, by measuring the secretion of cholesterol ester (CE)-rich β-very-low-density lipoprotein (β-VLDL).

To understand the abnormalities of lipid metabolism in ExHC rats, we analyzed various phenotypes of lipid metabolism in ExHC and SD rats. We investigated (1) the composition of Golgi lipoprotein (d < 1.006 g/mL), which is the composition of lipoproteins just before secretion from the liver in both ExHC and SD rats; (2) the activities of enzymes related to TAG metabolism (fatty acid synthase [FAS], Mg^2+^-dependent phosphatidic acid phosphohydrolase [PAP1], carnitine palmitoyltransferase [CPT], glucose 6-phosphate dehydrogenase [G6PDH] and malic enzyme); (3) the hepatic messenger RNA (mRNA) abundance related to lipid metabolism—acetyl-coenzyme A [CoA] acyltransferase (*Acat1*), apolipoprotein B (*Apob*), apolipoprotein E (*Apoe*), fatty acid translocase (*Cd36*), CPT on mitochondrial inner membrane (*Cpt1a*), *Cpt2*, diacylglycerol O-acyltransferase 1/2 (*Dgat1*/*2*), Δ5 fatty acid desaturase (*Fads1*), Δ6 fatty acid desaturase (*Fads2*), fatty acid synthetase (*Fasn*), glycerol kinase (*Gk*), glycerol-3-phosphate dehydrogenase 1 [soluble] (*Gpd1*), microsomal triglyceride transfer protein (*Mtp*), LDL receptor (*Ldlr*), hepatic lipase (*Lipc*), lipolysis-stimulated lipoprotein receptor (*Lsr*), phosphatidic acid phosphatase type 2A (*Ppap2a*), stearoyl-CoA desaturase (*Scd1*), acyl-CoA cholesterol O-acyltransferase (*Soat1*), sterol regulatory element binding transcription factor 1 (*Srebf1*), VLDL receptor (*Vldlr*); and (4) the fatty acid composition of CE in the serum and liver.

## Materials and methods

### Animals and diets

Male ExHC/Sea rat colonies have been maintained at our institute through brother-sister mating. Male 24-days-old SD/Sea rats were purchased from Seiwa Experimental Animal Co., LTD. (Fukuoka, Japan). For analysis of serum lipoprotein profile, male 24-days-old SD/Kud rats were purchased from KYUDO Co., LTD. (Saga, Japan). These animals had free access to a commercial nonpurified diet (NMF, Oriental Yeast Co., Tokyo, Japan) and deionized water and were maintained in a temperature-controlled room at 22–25°C with a 12-h light cycle (0800–2200 h). An experimental diet was formulated according to the AIN76™ formula [16] (Table 1). In our previous report, we affirmed that feeding a 1% cholesterol diet for 1 week induced hypercholesterolemia [17]. After consuming the experimental diet for 1 week (at 5 weeks old), the rats were killed via withdrawal of aortic blood under pentobarbitone anesthesia. The liver was immediately excised for use in subsequent experiments.

**Table 1:** Diet composition

**Ingredients**		**g/kg diet**
Sucrose		490
Casein		200
Corn starch		150
Mineral mixture (AIN76™)		35
Vitamin mixture (AIN76™)		10
Cholesterol		10
DL-methionine		3
Choline bitartrate		2
Olive oil		100
Fatty acids		
Palmitic acid	16:0	10.1
Palmitoleic acid	16:1	0.6
Stearic acid	18:0	3.8
Oleic acid	18:1	80.7
Linoleic acid	18:2	4.1
α-linolenic acid	18:3 n-3	0.4
Eicosenoic acid	20:1	0.3

The handling and killing of all animals were carried out in accordance with nationally prescribed guidelines, and ethical approval for the experiments was granted by the Animal Care and Use Committee, Kyushu University (Kyushu University, Scientific Research Promotion Division; authorization number: A22-160-2). In order to minimize suffering, we performed arterial blood collection under general anesthesia using subcutaneous sodium pentobarbitone (Kyoritsu Seiyaku Corporation, Tokyo, Japan), 0.08 mg/100 g body weight.

### Analysis of serum and liver parameters

Serum levels of cholesterol, TAG, non-esterified fatty acid (NEFA, free fatty acid), glucose, and free glycerol were measured with enzyme assay kits (Cholesterol C-Test, Triglyceride E-Test, NEFA C-Test, Glucose CII-Test: Wako Pure Chemical Industries, Osaka, Japan; Glycerol Assay Kit: Cayman Chemical Company, Ann Arbor, USA). Because amounts of glycerol generated via hydrolysis of serum TAG were measured with an assay kit (GPO-DAOS method [18]) to obtain serum TAG levels, collected serum TAG levels were calculated from measured TAG levels and serum free glycerol levels with the following formula:

Collected TAG level (mg/dL) = measured TAG level (mg/dL) – free glycerol level (mg/dL) × 885.4*/92.1** (*molecular weight of glycerol trioleate (triolein), **molecular weight of glycerol). Lipoprotein profiles of pooled blood serum obtained with the fast protein liquid chromatography method were analyzed using the Liposearch analytical lipoprotein profiling service (Skylight Biotech, Tokyo, Japan). Liver lipids were extracted using the method described by Folch et al. [19] and measured as total as well as free cholesterol, TAGs, and phospholipids (Phospholipid B-Test was also purchased from Wako Pure Chemical Industries).

### Analysis of Golgi VLDL

Isolation of structurally intact Golgi compartments was carried out using the method described by Swift et al. [20] with some modifications [21]. Intact Golgi pellets were resuspended in 1 mL of 154 mM NaCl and 15 mM Tris–HCl (pH 7.4). This resuspended fraction was rapidly passed through a Teflon homogenizer twice. Golgi VLDLs were floated through saline (d = 1.006) via ultracentrifugation for 16 h at 100,000 × *g* in a 100.2 Beckman rotor (Beckman Instruments, Palo Alto, CA, USA). Protein was measured using the method described by Lowry et al. [22]. Lipids were extracted from the lipoproteins as described by Folch et al. [19]. TAGs and phospholipids were determined as described elsewhere [23]. CE and free cholesterol were derivatized to trimethylsilyl ethers and quantified with gas–liquid chromatography on a 3% OV-17 column (GL Sciences, Tokyo, Japan) with 5α-cholestane (Nacalai Tesque, Kyoto, Japan) as an internal standard [24].

### Determination of hepatic mRNA levels

Total cellular RNA was isolated from liver tissue using a guanidinium thiocyanate/cesium chloride ultracentrifugation method according to Chirgwin et al. [25]. Complementary DNA was synthesized from 1.0 μg total RNA using a Transcriptor First Strand cDNA Synthesis Kit (Roche, Berlin, Germany). Expression levels for 23 genes related to lipid metabolism were analyzed using quantitative real-time reverse transcription polymerase chain reaction with a SYBR Premix EX Taq II kit and a Thermal Cycler Dice Real Time System TP800 (Takara, Shiga, Japan). The mRNA levels were normalized using the β-actin gene as an internal standard. Primer sequences for the analysis are shown in Table 2.

**Table 2:** **Primers for real**-**time RT**-**PCR**

**Gene**	**Forward primer**	**Reverse primer**	**Product (bp)**
Actb	TCAGGTCATCACTATCGGCA	TCATGGATGCCACAGGATTC	93
Mtp	CGACGGTGACGATGATCAACT	TGACCCGCATTTTCGACATT	66
Apob	ACAACCCTCACGGTCTTTGG	GAGACACGATCTGGAACTTG	171
Apoe	AGGAGCAGACCCAGCAGATA	GGAGTTGGTAGCCACAGAGG	143
Fasn	ATGCACACAGTGCTCAAAGG	GTATCCTCCACAGGCAGGAA	227
Scdl	GAGATACACTCTGGTGCTC	AAGGCGTGATGGTAGTTGTGG	156
Fads1	TCAGCGGAAGAAATGGGTG	GGATATGGTTCATCTGCGTCA	167
Fads2	TTGTCCTTGGAGAGTGGCAG	GTACATAGGGATGAGCAGCG	119
Dgat1	GATGCTCTTTTTCACCCAGC	GAGACGCTCAATGATTCGTG	118
Dgat2	CTGGCTGGCATTTGACTGGA	CTGGATGGGAAAGTAGTCTCG	108
Gk	GAATCCCACTCAGCCATTTG	TCCTAGAGCAGTTGTCTCGG	128
Gpdl	CAAACACCCAACTTCCGCATC	CAGCCCCAACAGCCACTATA	91
Ppap2a	GCGATGGCTACATTGAGAAC	GGCTTGAAGATAAAGTGCGAC	134
Srebf1	ATGCCATGGGCAAGTACACA	ACGTGTCAAGAAGTGCAAGG	179
Cptla	AAGGTGCTGCTCTCCTACCA	GGCCTCACAGATTCCAGGTA	193
Cpt2	TGACAGCCAGTTCAGGAGAA	ATACTCAGACTTTGGGTCCG	217
Soat1	GATGGGGTTATGTTGCTATGC	GGGCTCCTGTTTGATATTCCG	113
Acat1	CAGGTCTACCCATTGCCACT	CCCACCGTATGGTGTTGCTC	185
Ldlr	CACTGTGGCAGTAGTGAGTG	GGCTACCGTGAATACAGGAG	151
Vldlr	CCGTTCTACTCAGTGTATCCC	CGTCACAGTCATCCTGTCCA	176
Lipc	ACTCTTCCTCATCACCCGAG	CGCTGTTTTCCCACTTGAAC	257
Lsr	GAGGGTCCTATACTATATGGAG	TGGAGGGAGGTTACTTCACTC	108
Cd36	GAAGCACTGAAGAATCTGAAGAG	TCCAACACCAAGTAAGACCATC	159
Smek1	GAGCGACGGTTCTCTTCTTC	CAGACCACACAATCAGAGTGTC	86
Smek2	CTGCATATCAGAAGCAGCAG	ACTGATGGGTCCTTACCTTG	142

Gene symbols: *Acat1*, acetyl-CoA acyltransferase; *Apob*, apolipoprotein B; *Apoe*, apolipoprotein E; *Cpt1a*, CPT on mitochondrial outer membrane; *Cpt2*, CPT on mitochondrial inner membrane; *Dgat1*/*2*, diacylglycerol O-acyltransferase 1/2; *Fads1*, Δ5 fatty acid desaturase; *Fads2*, Δ6 fatty acid desaturase; *Fasn*, fatty acid synthetase; *Gk*, glycerol kinase; *Gpd1*, glycerol-3-phosphate dehydrogenase 1 (soluble); *Mtp*, microsomal triglyceride transfer protein; *Ppap2a*, phosphatidic acid phosphatase type 2A; *Scd1*, stearoyl-coenzyme A (CoA) desaturase; *Srebf1*, sterol regulatory element binding transcription protein 1; *Soat1*, acyl-CoA cholesterol acyltransferase. Uptake: *Cd36*, fatty acid translocase; *Ldlr*, low-density lipoprotein receptor; *Lipc*, hepatic lipase; *Lsr*, lipolysis-stimulated lipoprotein receptor; *Smek1*/*2*, homolog 1/2, suppressor of mek1 (*Dictyostelium*); *Vldlr*, very-low-density lipoprotein receptor.

### Determination of enzyme activity

Two grams of liver was homogenized in 6 volumes of a 0.25 M sucrose solution containing 1 mM ethylenediaminetetraacetic acid (EDTA) in 10 mM Tris–HCl buffer (pH 7.4). After precipitating the nuclei fraction, the supernatant was centrifuged at 10,000 × *g* for 10 min at 4°C to obtain mitochondria. The resulting supernatant was centrifuged again at 125,000 × *g* for 60 min at 4°C to precipitate microsomes, and the remaining supernatant was used as the cytosol fraction. The mitochondrial and microsomal pellets were resuspended in 0.25 M sucrose solution containing 1 mM EDTA in 10 mM Tris–HCl buffer (pH 7.4).

The activities of CPT in the liver mitochondrial fraction and Mg^2+^-dependent PAP1 in the liver microsomal fraction were determined as described by Markwell et al. [26], and by using a modified version [27] of the method described by Walton et al. [28], respectively. FAS activity was determined as described by Kelley et al. [29], G6PDH activity was measured using the method described by Kelley et al. [30], and the activity of malic enzyme in the liver cytosol fraction was determined as described by Ochoa [31]. Activities of β-oxidation in the liver peroxisomal fraction were measured using the method described by Lazarow [32]. Serum lecithin:cholesterol acyltransferase (LCAT) activity was measured with enzyme assay kits (ANASOLV® LCAT, SEKISUI Medical Co. LTD., Tokyo, Japan) [33].

### Analysis of fatty acid compositions of serum and liver CE

Lipid samples extracted from the serum and the liver were separated with thin-layer chromatography, and CE tractions were obtained. Fatty acid composition was analyzed using gas–liquid chromatography (GC8A, Shimadzu, Kyoto, Japan) on an Omegawax 320 capillary column (Supelco, Japan, Tokyo) as described elsewhere [34]. Pattern similarities of fatty acid composition between serum and liver CE were calculated. Pattern similarity was defined as “the index of product sum of components standardized by square roots of sum of squares of each component, when 2 samples are regarded as vectors (A = [a_1_, a_2_, …, a_n_], B = [b_1_,b_2_, …, b_n_])” and calculated with the following:

$$\mathrm{Pattern}\;\mathrm{similarity}=\mathrm{cos\theta}=\frac{\sum_{1}^{n}a_{i}b_{i}}{\sum_{1}^{n}a_{i}^{2}\sum_{1}^{n}b_{i}^{2}}$$

### Statistical analysis

Data for each measurement (n = 5/group) were analyzed through inspection of all differences using the Student’s *t*-test with Excel 2010 (Microsoft, Redmond, USA) and the add-in software Statcel 3. Differences were considered significant when p was <0.05.

## Results

### Growth parameters

As the result of matching in age, initial body weights, and hence final body weights of ExHC rats were significantly lower than those of SD rats (Table 3). Body weight gain, total food intake, food efficiency, relative liver weight were similar between the strains (Table 3). Judging from the similar total food intake, total cholesterol intakes were similar between the strains.

**Table 3:** Growth parameters

	**SD**	**ExHC**
Initial body weight (g)	113.5 ± 4.2	95.8 ± 2.5**
Final body weight (g)	163.6 ± 2.1	143.6 ± 3.2**
Body weight gein (g)	50.2 ± 3.7	47.9 ± 4.1
Food intake (g)	97.0 ± 0.0	97.2 ± 3.3
Food efficiency (g body weight gein/g food intake)	0.517 ±0.038	0.492 ± 0.038
Liver weight (g/100 g body weight)	4.42 ± 0.13	4.56 ± 0.07

Values are mean ± standard error of the mean (SEM); n = 5. **p < 0.01. ExHC, exogenously hypercholesterolemic; SD, Sprague–Dawley.

### Serum and liver parameters and serum lipoprotein profile

**Figure 1:**
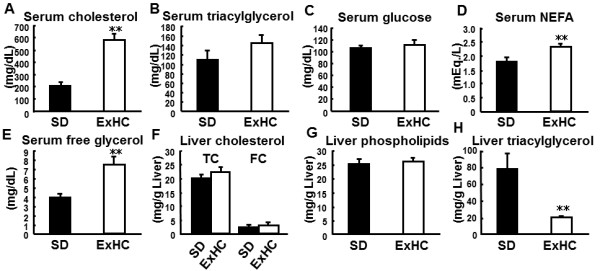
**Serum and liver parameters in rats. (A–E)** Serum parameters (**A**: total cholesterol; **B**: triacylglycerol [TAG]; **C**: glucose; **D**: non-esterified fatty acid [NEFA]; and **E**: free-glycerol) in Sprague–Dawley (SD/Sea; solid bar) and exogenously hypercholesterolemic (ExHC; open bar) rats were measured with enzyme assay. Rats were fed a cholesterol-containing diet for 1 week. In **(B)**, serum TAG levels represent collected TAG levels calculated with the formula given in the Analysis of Serum and Liver Parameters section in the Materials and Methods. **(F–H)** Liver lipids (**F**: total and free cholesterol; **G**: TAG; **H**: phospholipids) in SD (solid bar) and ExHC (open bar) rats were extracted using the method described by Folch et al. [19] and measured with enzyme assays. TC and FC in **(F)** refer to total cholesterol and free cholesterol, respectively. Values are mean ± standard error of the mean (SEM); n = 5; **p < 0.01.

As shown in Figure 1, serum cholesterol levels in ExHC rats were higher than those in SD rats, whereas ExHC and SD rats showed similar levels of liver total and free cholesterol. Conversely, ExHC and SD rats showed similar serum TAG levels, whereas ExHC rats had lower liver TAG levels. ExHC and SD rats did not differ in serum glucose and liver phospholipid levels. Serum NEFA and free glycerol levels in ExHC rats were significantly higher than those in SD rats.

**Figure 2:**
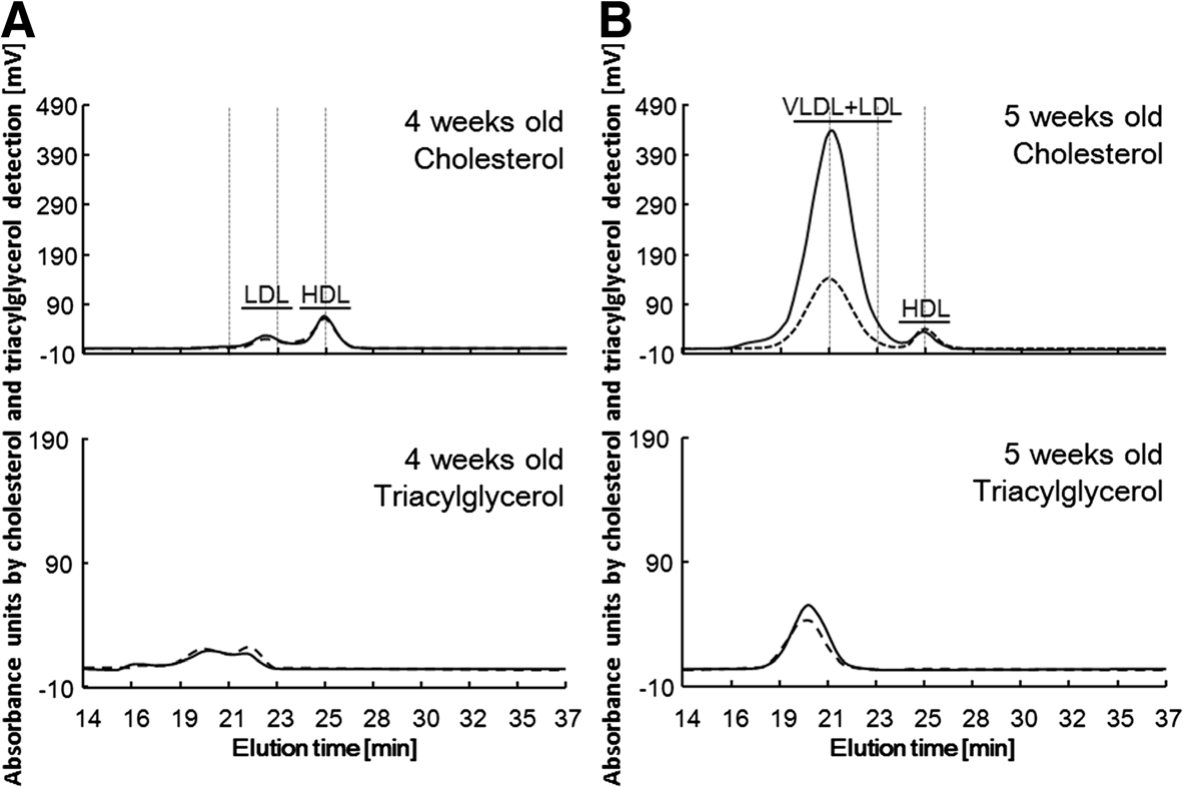
**Serum lipoprotein profiles of cholesterol and triacylglycerol.** Cholesterol (upper) and TAG (lower) profiles in fast protein liquid chromatography fractions of pooled blood serum from SD/Kud (dash line) and ExHC (solid line) rats **(A)** at 4 weeks of age (before being fed a cholesterol-containing diet) and **(B)** at 5 weeks of age (after being fed the cholesterol-containing diet for 1 week).

The serum lipoprotein profile in SD and ExHC rats are shown in Figure 2. These profiles were similar in the 2 strains before the cholesterol-containing diet was fed (at 4 weeks of age). By contrast, ExHC rats showed higher cholesterol levels in the VLDL + LDL fraction (mainly VLDL) after consuming a cholesterol-containing diet (at 5 weeks of age).

### Composition of lipoprotein (d < 1.006) secreted from Golgi apparatus

**Figure 3:**
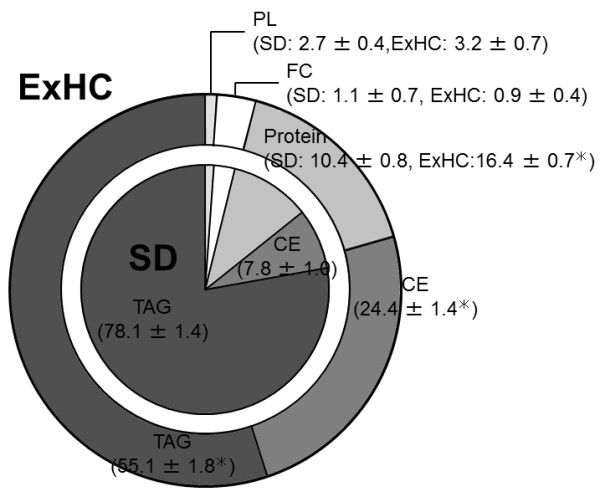
**Composition (wt%) of Golgi lipoprotein (d < 1.006) from SD and ExHC rats.** Composition (wt%) of Golgi lipoprotein (d < 1.006) from SD/Sea (inside) and ExHC (outside) rats was analyzed using the Lowly method (proteins) and gas–liquid chromatography (lipids). Values are means ± SEM; n = 5. *p < 0.05. CE, cholesterol ester; FC, free cholesterol; PL; phospholipid; TAG, triacylglycerol.

Although the proportions of free cholesterol and phospholipids in lipoprotein (d < 1.006) secreted from Golgi apparatus did not differ between strains, the proportions of CE and protein were increased and TAG was decreased in ExHC rats (see Figure 3). Therefore, the ratio of CE to TAG in lipoprotein in ExHC rats was 4 times that in SD rats (SD: 0.1 ± 0.0; ExHC: 0.4 ± 0.0; p < 0.05).

### mRNA expression of lipid metabolism–related genes in the liver

**Figure 4:**
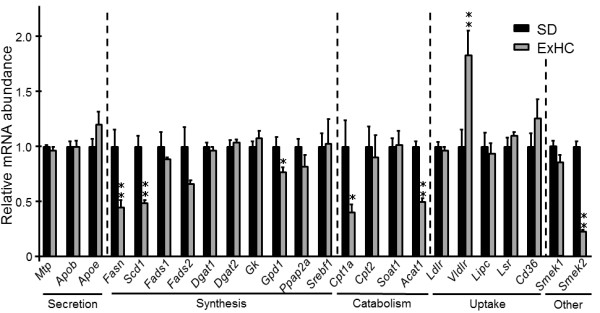
**Hepatic messenger RNA expression levels in SD and ExHC rats.** Hepatic messenger RNA (mRNA) expression levels in SD/Sea and ExHC rats were measured using real-time reverse transcription-polymerase chain reaction. The β-actin gene was used as an internal control, and data were set relative to SD rats. Values are means ± SEM; n = 5. *p < 0.05; **p < 0.01. See Table 2 for a key to the abbreviations and sequences of the primers used.

Figure 4 shows the mRNA expression of lipid metabolism–related genes in the liver. Genes related to lipid secretion—*Mtp*, *Apob*, and *Apoe*—in the liver of ExHC rats were expressed at the same levels as those in SD rats. mRNA levels of *Fasn* and *Scd1* in ExHC rats were significantly lower than those in SD rats, and that of *Fads2* also tended to be lower (p = 0.09) in ExHC rats, but the expression of some genes related to TAG and fatty acid synthesis—*Fads1*, *Dgat1*/*2*, *Gk*, *Ppap2a*, *Srebf1* —were similar between the 2 strains. Furthermore, the mRNA level of *Gpd1* was decreased in ExHC rats. In gene expression related to TAG and fatty acid catabolism, *Cpt1a* and *Acat1* mRNA levels were significantly decreased in ExHC rats. *Cpt2* and *Soat1* mRNA levels did not differ between strains. In a group of genes related to lipid uptake by the liver, the level of *Vldlr* mRNA in ExHC rats was twice that in SD rats. *Lipc*, *Ldlr* (LDL receptor), *Lsr*, and *Cd36* mRNA expression levels were similar between the 2 strains.

### Activities of serum LCAT and enzymes related to TAG metabolism in the liver

As shown in Table 4, FAS activity in the hepatic cytosolic fraction of ExHC rats was significantly lower than that in SD rats. The activity of CPT in ExHC rats was higher than that in SD rats. The activity of peroxisomal β-oxidation of fatty acids in ExHC rats was lower than that in SD rats. No differences in malic enzyme activity, G6PDH in the hepatic cytosolic fraction, PAP1 in the hepatic microsomal fraction, or serum LCAT occurred between the 2 strains.

**Table 4:** Activities of hepatic enzymes and serum LCAT activity in SD and ExHC rats

	**SD**	**ExHC**	
Liver enzyme activities	*nmol* mn^-1^ mg^-1^*protein*		
Fatty acid synthase	37.8 ± 2.9	29.5 ± 2.1*	
Malic enzyme	83.2 ± 6.1	81.6 ± 10.1	
Glucose 6-phosphate dehydrogenase	90.9 ± 10.0	87.7 ± 18.4	
Phosphatidate phosphohydrolase			
Mg^2+^-dependent	17.4 ± 0.6	15.7 ± 1.1	
Carnitine palmitoyl transferase	1.42 ± 0.13	2.52 ± 0.21*	
Peroxisomal β-oxdation	9.40 ± 1.39	3.92 ± 0.75*	
Serum enzyme activity	*Unit*^┼^		
LCAT	268 ± 22	251 ± 39	

Values are mean ± SEM; n = 5. *p < 0.05. ExHC, exogenously hypercholesterolemic; SD, Sprague–Dawley.

^✝^Unit of lecithin:cholesterol acyltransferase (LCAT) activity is defined as a concentration of esterified free-cholesterol at 37°C in 1 h in the operating manual of the ANASOLV® LCAT (SEKISUI Medical Co. LTD., Japan).

*Abbreviations*: *LCAT* lecithin:cholesterol acyltransferase.

### Fatty acid composition of serum and liver CE

**Figure 5:**
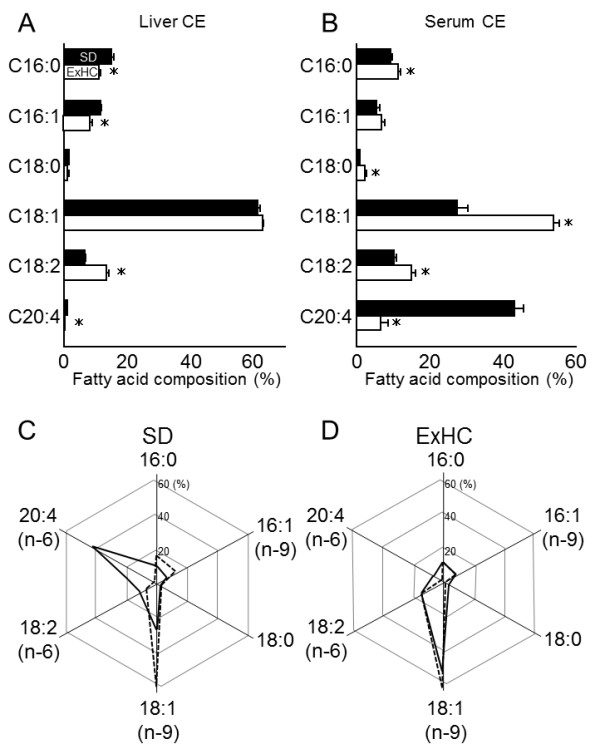
**Fatty acid composition of cholesterol ester in serum and liver of SD and ExHC rats. (A,B)** Fatty acid composition of cholesterol ester (CE) in **(A)** liver and **(B)** serum of SD/Sea (solid bar) and ExHC (open bar) rats was analyzed with gas chromatography. Values are means ± SEM; n = 5. *p < 0.05. **(C,D)** Comparison of fatty acid composition between liver CE (dashed line) and serum CE (solid line) in **(C)** SD rats and **(D)** ExHC rats.

In the fatty acid composition of serum CE, proportions of palmitic acid, stearic acid, oleic acid, and linoleic acid were increased in ExHC rats compared with those in SD rats, and the proportion of arachidonic acid was decreased in ExHC rats. Conversely, in liver CE, the proportion of linoleic acid was increased and proportions of palmitic acid, palmitoleic acid, and arachidonic acid were decreased in ExHC rats (see Figure 5A,B).

The pattern similarity of the fatty acid composition in hepatic and serum CE in ExHC rats had a higher score at 0.9919 than that in SD rats at 0.5767. Pattern similarity between the hepatic CE fatty acid compositions in both strains was 0.9911 (see Figure 5C,D).

## Discussion

After being fed a cholesterol-containing diet, ExHC rats developed hypercholesterolemia and displayed low liver TAG content (see Figure 1). This hepatic TAG reduction was due to reduced enzyme activities such as those of FAS (see Table 4) related to fatty acid synthesis as a result of decreasing mRNA levels (see Figure 4). This reduction of liver TAG content led to a decline in the proportion (wt%) of TAG in all constituents of lipoprotein (d < 1.006) newly synthesized by Golgi apparatus (see Figure 3). We have reported that the amount of cholesterol secreted by the liver in ExHC rats is identical to that secreted by the livers of SD rats [17]. Taken together, the CE/TAG ratio in VLDL of ExHC rats significantly increases with decreases in hepatic TAG secretion, and the lipoprotein secreted by ExHC rats is a CE-rich VLDL (defined as β-VLDL). Hepatic intake of β-VLDL is delayed in ExHC rats because the affinity of β-VLDL for LDL receptors in ExHC rats is low [34]. In ExHC rats, this delay slows catabolism and β-VLDL retention and causes a sequential increase in serum total cholesterol levels. This process is the pathogenic mechanism of hypercholesterolemia in ExHC rats, and this serum cholesterol–regulated mechanism is a novel one that has not been previously reported. In ExHC rats, hepatic TAG decrease leads to serum cholesterol elevation only when cholesterol is included in the diet. Furthermore, we have previously reported that a high linoleic safflower oil- and cholesterol-containing diet increases hepatic TAG levels and ameliorates hypercholesterolemia in ExHC rats compared with the effects of an olive oil– and cholesterol-containing diet [35]. These changes related to differences in dietary fatty acids were not observed in SD rats. These data also support an inverse relationship between hepatic TAG levels and hypercholesterolemia in ExHC rats.

Hepatic TAG levels are regulated by (1) quantitative and qualitative intake of dietary TAG, (2) TAG synthesis, (3) catabolism, and (4) VLDL secretion. In a comparison of mRNA abundance in the liver in ExHC and SD rats, the lower expression levels of genes for fatty acid synthesis were particularly important. In addition, FAS activity was lower, and mRNA levels of *Scd1*, a modification enzyme for fatty acids, were decreased in ExHC rats. *Srebf1* codes SREBP-1c which is one of transcriptional factors and regulates FAS mRNA [36], its mRNA expression levels were similar between the 2 strains (see Figure 4). The behavior of SREBP-1c in cells are regulated by many post-translational modification of phosphorylation and ubiquitination, and so on [37,38]. Judging from these informations, the decreases in mRNA levels of genes related to *de novo* fatty acid synthesis in ExHC rats might be a result of hypoactive SREBP-1c behavior. The decreases in FAS activities suggested that *de novo* fatty acid synthesis is lower in ExHC rats, decreasing TAG synthesis. Moreover, the mRNA level of glycerol-3-phosphate dehydrogenase, which supplies glycerol-3-phosphate as a TAG synthesis substance, was lower in ExHC rats. *De novo* fatty acids and glycerol-3-phosphate are derived from glucose. These data suggested that glucose metabolism in ExHC rats is abnormal. *Smek2*, identified as a gene responsible for hypercholesterolemia induced by dietary cholesterol in ExHC rats, reportedly involves gluconeogenesis [13]. Another determinant of hepatic TAG levels is fatty acid catabolism. In ExHC rats, peroxisomal β-oxidation was significantly lower, and mitochondrial CPT activities were significantly higher compared with those of SD rats (Table 4). The decrease in peroxisomal β-oxidation, which mainly shortens long-chain (>C18:0) fatty acids, may reflect a decline in long-chain fatty acids, as demonstrated by the low proportion of C20:4 in hepatic CE (Figure 5A). The increase in mitochondrial CPT activities implied a high level of mitochondrial β-oxidation. Therefore, along with the low FAS activities, high mitochondrial β-oxidation may decrease hepatic TAG content in ExHC rats.

Cholesterol metabolism has been analyzed in detail in ExHC rats [14,15,17,39]. Cholesterol intestinal absorption [14], hepatic synthesis [39], and excretion in feces [15] in ExHC rats are similar to those in SD rats. We have also reported that when ExHC rats are fed a cholesterol diet, the uptake of blood β-VLDL by the liver is lower in ExHC rats compared with that in SD rats fed a cholesterol diet because both the association and the degradation of β-VLDL by the liver cells is lower in ExHC rats [17]. In this study, the mRNA levels of *Ldlr* and *Lsr* in ExHC rats did not differ from those in SD rats (see Figure 4). *Cd36* and *Lipc* mRNA levels, which relate to fatty acid uptake, were also not different. Among genes for which expression levels were measured, the mRNA level of *Vldlr* in ExHC rats was significantly higher than that of SD rats. Although the mechanism is unclear, serum VLDL reportedly upregulates *Vldlr* mRNA expression in a macrophage cell line [40]. The increase of *Vldlr* mRNA level seems to be due to the increase of VLDL particles or chemical components of β-VLDL. In other words, the increase of VLDL particles in ExHC rats might increase *Vldlr* mRNA levels. Additionally, the amount of LDL receptor protein in the liver was similar in the 2 strains [15]. Briefly, the delay in hepatic β-VLDL uptake was not caused by the amounts of receptor proteins and mRNA. Huff et al. [41] have reported that β-VLDL has an abundance of not only CE but also apolipoprotein E (apoE). ApoE-rich β-VLDL shows high affinity for LDL receptors and is taken up by liver cells [42]. However, we have shown that serum apoE levels are similar between ExHC and SD rats fed cholesterol [15]. Despite the β-migration of VLDL caused by low TAG synthesis in ExHC rats, serum apoE levels were not higher, which delayed hepatic uptake of β-VLDL in ExHC rats.

In order to obtain the evidence of delayed β-VLDL catabolism in ExHC rats, we investigated the fatty acid composition of serum and hepatic CE. The hepatic and serum CE fatty acid compositions in SD rats were dissimilar. We calculated pattern similarity between these compositions with a value of 0.5767 in these rats (see Figure 5C). However, hepatic and serum fatty acid compositions in ExHC rats were highly similar and displayed a value of pattern similarity of 0.9919 (see Figure 5D). Moreover, we calculated pattern similarity between hepatic CE fatty acid compositions in both strains, and the value was 0.9911. These values imply that ExHC and SD rats have highly similar hepatic CE fatty acid compositions, and only SD rats display modified CEs after secretion. The CE pool in the lipoprotein particles in SD rats may frequently undergo fatty acid transport by LCAT in the circulation. However, serum LCAT activities were the same in the 2 strains (see Table 4). The high CE/TAG ratio in the serum lipoprotein of ExHC rats might inhibit the modification of CEs by LCAT in the circulation.

We measured serum glucose and free glycerol levels. Although serum glucose levels were similar between the 2 strains, serum free glycerol levels in ExHC rats were significantly higher than those in SD rats (see Figure 1C, E). Free glycerol in blood derives from lipolysis in the blood by lipoprotein lipase and in adipose tissues via hormone-sensitive lipase. Free glycerol is taken up by the liver as a substrate of gluconeogenesis and TAG synthesis. Glycerol metabolism is mediated by insulin, which suppresses glycerol emission from adipose tissues and glycerol uptake by the liver [43]. However, Kiriyama et al. [44] have reported that glycerol metabolism is not suppressed in db+/db + mice despite their hyperinsulinemia. Moreover, lipolysis in adipose tissue and gluconeogenesis in the liver are facilitated in these mice. The abnormality in glycerol metabolism in ExHC rats may involve an increase in serum NEFA levels and a decrease in hepatic mRNA expression of *Gpd1* encoding glycerol-3-phosphate dehydrogenase 1. Glycerol is supplied and catabolized in glucose metabolism. Also, NEFA behavior in serum relates to glucose metabolism. Additionally, it has been reported that SMEKs (SMEK1 and SMEK2) positively regulate hepatic gluconeogenesis via dephosphorylation of cyclic adenosine monophosphate–response element binding protein-regulated transcriptional coactivator 2 as regulatory subunits of protein phosphatase 4 [13]. We hypothesize that the elevation in serum cholesterol levels in ExHC rats is caused by a decrease in hepatic TAG synthesis, which may be caused by delayed glucose metabolism controlled by *Smek2*. Further investigation of glucose metabolism in ExHC rats is needed.

**Figure 6:**
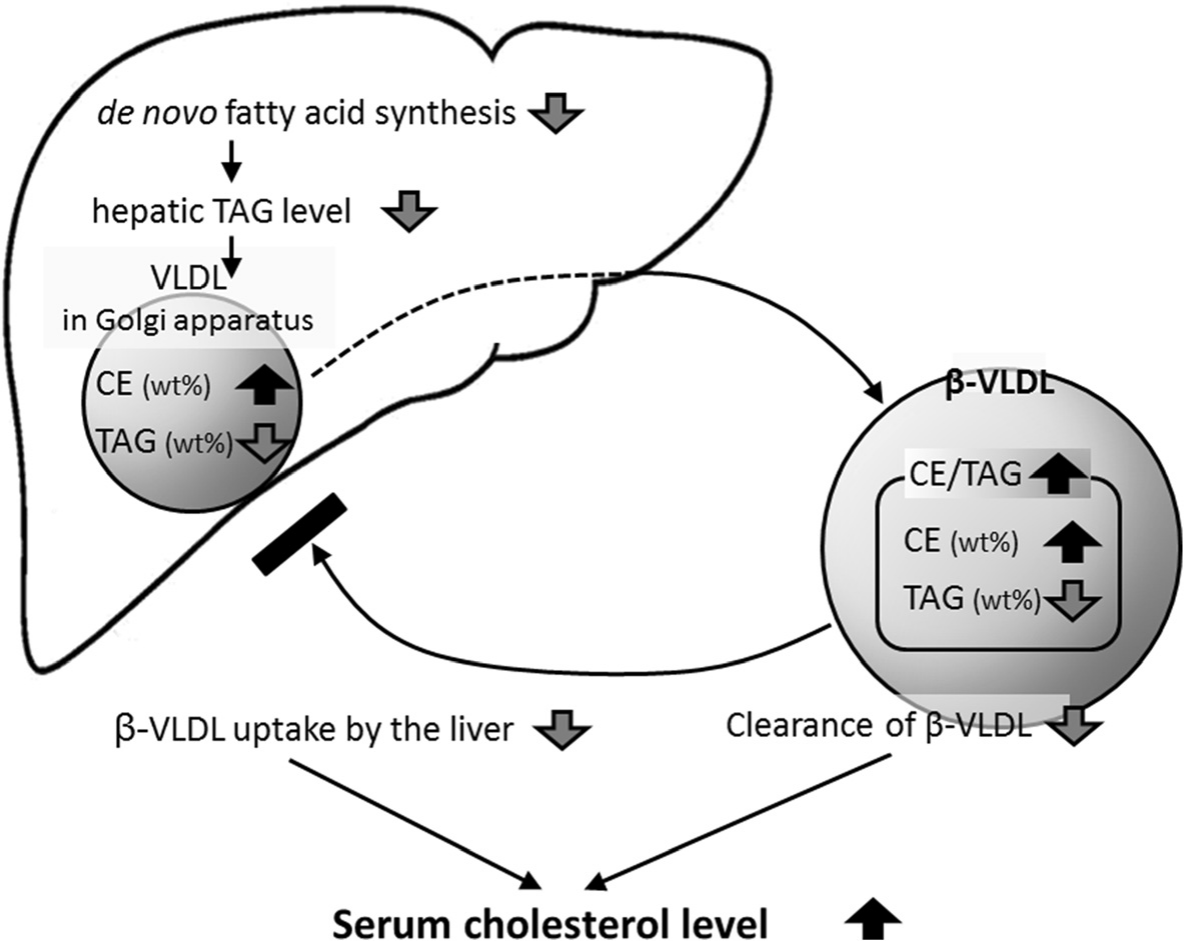
**Pathogenic mechanism of hypercholesterolemia in ExHC rats.** Directional lines represent chronology of events. Black and gray thick arrows represent increases and decreases in phenotypes of ExHC rats compared with those of SD rats, respectively. TAG and CE refer to triacylglycerol and cholesterol ester, respectively.

In conclusion, we illustrated the pathogenic mechanism of hypercholesterolemia in ExHC rats (Figure 6). This study demonstrates that decreases in hepatic TAG synthesis in ExHC rats leads to hepatic secretion of CE-rich, TAG-poor β-VLDL. The production of these abnormal particles depends on decreases in hepatic fatty acid and TAG syntheses. In addition, the similarity in fatty acid composition between serum and hepatic CE in ExHC rats indicates that the behavior of circulatory CE is unaffected by modification. To gain insights into these observations, we must grasp glycometabolism in ExHC rats and investigate the involvement of *Smek2* in lipid metabolism.

## Abbreviations

apoE: Apolipoprotein E; CE: Cholesterol ester; CoA: Coenzyme A; CPT: Carnitine palmitoyltransferase; EDTA: Ethylenediaminetetraacetic acid; ExHC: Exogenously hypercholesterolemic; FAS: Fatty acid synthase; G6PDH: Glucose 6-phosphate dehydrogenase; LCAT: Lecithin:cholesterol acyltransferase; NEFA: Non-esterified fatty acid; PAP: Phosphatidic acid phosphohydrolase; SD: Sprague–Dawley; SDS: Sodium dodecyl sulfate; SREBP: Sterol regulatory element-binding protein; TAG: Triacylglycerol; VLDL: Very-low-density lipoprotein.

## Competing interests

The authors of the manuscript declare no conflicts of interest.

## Authors’ contributions

YT, KI, MS designed the research; YT, KN, HN, TN, YI, HM, MA, MS performed the research; YT, KN, HM, MA, MS analyzed data; YT, KI, MS drafted the manuscript; All authors read and approved the final manuscript.
